# Water stress memory in wheat/maize intercropping regulated photosynthetic and antioxidative responses under rainfed conditions

**DOI:** 10.1038/s41598-023-40644-1

**Published:** 2023-08-22

**Authors:** Sadam Hussain, JinJin Wang, Muhammad Asad Naseer, Muhammad Saqib, Manzer H. Siddiqui, Fahid Ihsan, Chen Xiaoli, Ren Xiaolong, Saddam Hussain, Hafiz Naveed Ramzan

**Affiliations:** 1 College of Agronomy, Key Laboratory of Crop Physio-Ecology and Tillage in Northwestern Loess Plateau, Ministry of Agriculture, Northwest A&F University, Yangling, Shaanxi China; 2 Key Laboratory of Agricultural Soil and Water Engineering in Arid and Semi-Arid Area, Ministry of Education/Institute of Water Saving Agriculture in Arid Areas of China, Northwest A&F University, Yangling, Shaanxi China; 3 Barani Agricultural Research Station, Fateh Jang, Attock, Punjab 43350 Pakistan; 4 Department of Botany and Microbiology, College of Science, King Saud University, Riyadh, 11451 Saudi Arabia; 5 Agronomic Research Institute, Ayub Agricultural Research Institute, Faisalabad, Punjab Pakistan; 6 Plant Stress Physiology Lab, Department of Agronomy, University of Agriculture, Faisalabad, 38040 Pakistan

**Keywords:** Photosynthesis, Plant development, Plant physiology, Plant stress responses

## Abstract

Drought is a most prevalent environmental stress affecting the productivity of rainfed wheat and maize in the semiarid Loess Plateau of China. Sustainable agricultural practices such as intercropping are important for enhancing crop performance in terms of better physiological and biochemical characteristics under drought conditions. Enzymatic and non-enzymatic antioxidant enzyme activities are associated with improved abiotic tolerance in crop plants, however, its molecular mechanism remains obscure. A 2-year field study was conducted to evaluate the influence of intercropping treatment viz. wheat mono-crop (WMC), maize mono-crop (MMC), intercropping maize (IM) and wheat (IW) crops, and nitrogen (N) application rates viz. control and full-dose of N (basal application at 150 and 235 kg ha^−1^ for wheat and maize, respectively) on chlorophyll fluorescence, gas exchange traits, lipid peroxidation, antioxidative properties and expression patterns of six tolerance genes in both crops under rainfed conditions. As compared with their respective monocropping treatments, IW and IM increased the Fo/Fm by 18.35 and 14.33%, PS-11 efficiency by 7.90 and 13.44%, photosynthesis by 14.31 and 23.97%, C-capacity by 32.05 and 12.92%, and stomatal conductance by 41.40 and 89.95% under without- and with-N application, respectively. The reductions in instantaneous- and intrinsic-water use efficiency and MDA content in the range of 8.76–26.30% were recorded for IW and IM treatments compared with WMC and MMC, respectively. Compared with the WMC and MMC, IW and IM also triggered better antioxidant activities under both N rates. Moreover, we also noted that intercropping and N addition regulated the transcript levels of six genes encoding non-enzymatic antioxidants cycle enzymes. The better performance of intercropping treatments i.e., IW and IM were also associated with improved osmolytes accumulation under rainfed conditions. As compared with control, N addition significantly improved the chlorophyll fluorescence, gas exchange traits, lipid peroxidation, and antioxidant enzyme activities under all intercropping treatments. Our results increase our understanding of the physiological, biochemical, and molecular mechanisms of intercropping-induced water stress tolerance in wheat and maize crops.

## Introduction

Intensive agricultural practices such as monocropping, deep tillage, and chemical fertilizers application are the major drivers of global climate change, which affects crop cultivation and productivity^1,2^. Under variations of the climatic variables, environmental stresses such as drought and extreme light conditions cause severe losses to crop growth, development, and overall yield^3,4^. Besides elevated evapotranspiration rates, precipitation is pretty less inevitable and extreme under increasing temperatures in several parts of the globe^5^. In addition to variable climatic conditions, startling population growth rates are also among the critical challenges for future food security^6,7^. These conditions exert a huge pressure on existing lands to produce more food for the frightening global population^8^. On the other hand, existing cultivated land is lessening at a fast pace under the era of industrialization and urbanization; thus, it is imperative need to produce more yield from the existing farmland area by adapting sustainable agronomic approaches.

Intercropping is one of the important sustainable approaches adopted throughout the world because of its positive influence on crop productivity^9^, yield stability^10,11^, and nutrients and water use efficiency^12,13^. The improved and stabled yields in the intercropping system are mainly associated with the circumstance of “growth recovery”^14^. In intercropping system, the dominant crop gets more advantages in terms of used nutrients and soil moisture, and ultimately in growth and productivity than the inferior crop^15^. Nonetheless, after harvesting the dominant crop, a rapid increase in growth and development of inferior crops has been observed because of less competition for available resources^16^. Intercropping is adopted throughout China including the Loess Plateau region which covers about 45% of the country’s cultivated area under winter wheat^17,18^. The Loess Plateau in China covers about 65 million hectares and provides livelihood to millions of the population^19^. The Loess Plateau, located in the semi-arid region of northwest China, is one of the most significant eco-fragile regions with scarce water resources, sparse vegetation, and poor land productivity. This region is experienced severe dry periods due to limited rainfall and high evaporation^20^. In this region, the rainfall is primarily concentrated only between July and September. Thus, in these areas, the frequency of drought episodes is likely to exacerbate in the forthcoming eras of multifactorial stress induced by climate change^21^. Limited soil moisture conditions affected the growth, development, and overall yield of field crops including wheat and maize^22^. Indeed, improved WUEs are highly dependent on enhanced soil water consumption during the crop growth period. Consequently, decreasing evapotranspiration and increasing the conservation of rainwater through sustainable agricultural management practices are crucial for increasing crop productivity in rainfed areas.

Water-scarce conditions impede plant growth, development, and ultimately the productivity of field crops^23,24^. Prolonged drought impacted various plant components including metabolic, morphophysiological and biochemical events in major field-grown crops^25,26^. Previously, some studies have demonstrated that soil water shortage induces a negative impact on cell water potential, results in stomatal closure and reduces the efficiency of photosynthetic machinery, and decreases nitrate assimilation in crop plants^27,28^. Drought also resulted in the excessive accumulation of reactive oxygen species (ROS) such as the superoxide radical (O_2_) and hydrogen peroxide (H_2_O_2_) which in turn causes oxidative stress by triggering membrane injuries^29^, lipid and protein degradation^30^, inactivation of numerous enzymes^31^ and reducing the accumulation of antioxidative enzymes^32^. To alleviate ROS accumulation under stress conditions, numerous enzymatic antioxidants such as superoxide dismutase (SOD), catalase (CAT), glutathione reductase (GR), and ascorbate peroxidase (APX) are accumulated in plants. Moreover, some non-enzymatic antioxidants such as glutathione (GSH) and ascorbate (ASA) also play a role in detoxifying the ROS. The SOD catalyzes the dismutation of O_2_ to oxygen (O_2_) and H_2_O_2_, which is subsequently reduced to H_2_O and O_2_ by CAT, APX, GR, etc.^33^. Ascorbate and GSH work as cofactors of enzymes of the antioxidant pathways, both can also directly quench ROS. In recent years, most published studies have evaluated the impact of drought on physiological and biochemical traits under controlled conditions. Nonetheless, only a limited number of studies are available on the influence of drought on aforementioned traits under field conditions. Most importantly, little information is available on the performance of maize/wheat intercropping and its effects on the physiological and biochemical characteristics of both crops grown under rainfed conditions. Hence, a 2-year field experiment was conducted to evaluate the mechanism of water stress adaptation in maize/wheat intercropping. Comparative performance of maize/wheat intercropping with regards to chlorophyll fluorescence, antioxidative defense, lipid peroxidation, and regulation of stress responsive genes under rainfed conditions was evaluated. Furthermore, variations in physiological and biochemical traits of intercropping rows were investigated under different N rates. It was hypothesized that wheat–maize intercropping may perform better in terms of physiological, biochemical, and molecular traits when compared with monocropping under rainfed water-scarce conditions.

## Materials and methods

### Plant guideline/accordance statement

All the methods included in this study were performed in accordance with the relevant guidelines and regulations.

### Plant material

The seeds of local popular wheat and maize cultivars viz. Yongliang 4 and Xianyu 335 were collected from the Northwest A&F University and used with seedling rates of 180 kg ha^−1^ and 66,670 maize plants ha^−1^, respectively. For wheat crop, a 20 cm inter-row spacing both for intercropping and monoculture was maintained. A 50 cm inter-row spacing and 30 cm intra-row spacing, same spacing both for intercropping and monoculture, was kept for maize crop.

### Experimentation

**Figure 1:**
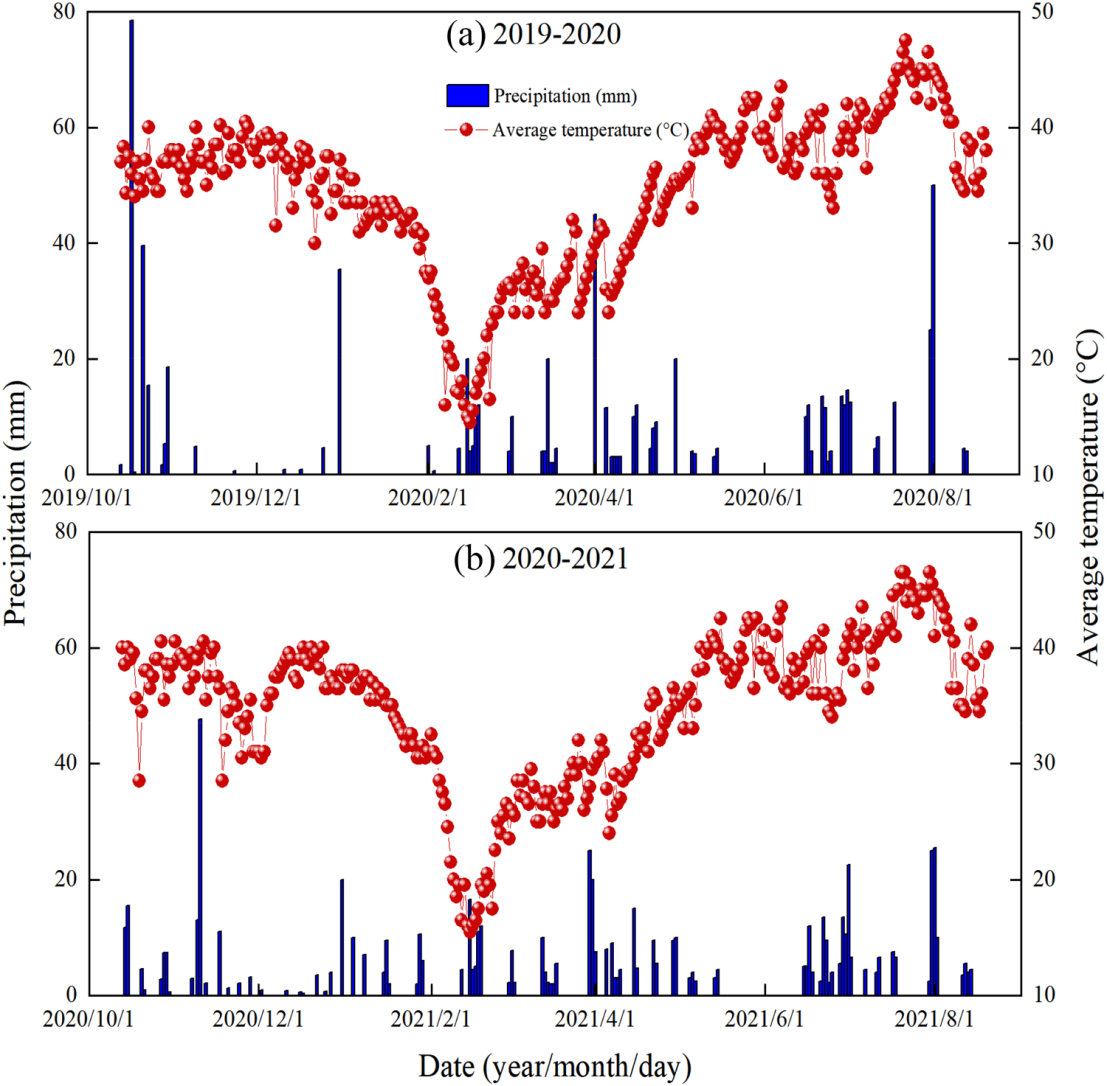
Daily weather data including the precipitation and average temperature during both experimental years.

To explore the influence of maize/wheat intercropping on plants’ physiological and biochemical events under rainfed conditions, a 2-year field experiment was conducted at Northwest A&F University for two consecutive years (2019 and 2020). The study site has loam soil with > 25% field capacity. During the last four years, the experimental field had been under the cultivation of spring maize crop. Likewise, the study site had the following climatic properties: 14.5 °C and about 500 mm of mean annual temperature and mean annual precipitation, respectively; of which > 70% of rainfall has occurred only between the months of July and September thus crops are frequently exposed the various episodes of water stress (Fig. 1). The soil (above 0–30 cm layer) before the start of the experiment had the following properties: soil pH of 8.12, and 0.8792, 0.0490, 0.0149, and 0.0954 g kg^−1^ of total nitrogen-N, available N, phosphorus, and potash, respectively. The experimental units were arranged with a randomized complete block design in a split-plot arrangement with three replicates, on the research field of water/moisture stress area. The treatments evaluated were maize monocrop (MM), wheat monocrop (WM), intercropping maize (IM), and wheat (IW) crops under two N application rates: control (without N) and a full dose of N (basal application at 150 and 235 kg N ha^−1^ for wheat and maize, respectively, for both mono- and intercrops). The total numbers of experimental units were 18. Each experimental unit was 10.5 m in length and 9 m in width and there was 1 m buffer zone between adjacent plots. In relay strip intercropping system, three complete wheat/maize intercropping strips formed a plot. Each strip consisted of eight rows of wheat plants (strip 1.6 m wide) and four rows of maize plants (strip 1.9 m wide). Therefore, 45.7% of the land area in each intercropped plot was occupied by wheat whereas the remaining 54.3% was covered by maize crop. Wheat was sown on October 21, 2019, and October 13, 2020, and maize was sown on April 06, 2020, and March 30, 2021, during the first and second experimental years, respectively. Winter wheat was harvested on June 20, 2020, and 2021, and spring maize was harvested on August 24, 2020, and August 01, 2021. The competitive growth phase between the two crops was about 2 months during both years. Based on the site recommended, phosphorus and potassium were applied at 176 and 40 kg ha^−1^ by using tricalcium phosphate {Ca_3_(PO_4_)_2_} and sulphate of potash, respectively. All fertilizers, including treated N, were applied as the basal dose of both crops under both monocropping and intercropping treatments. No irrigation was applied during the experimentation.

### Observations and measurements

#### Chlorophyll fluorescence and gas exchange

Crops were harvested at the physiological maturity stages of both crops. Young fully expanded leaves of both crops were considered for measuring the chlorophyll fluorescence (CF) and gas exchange attributes by using a portable multifunction photosynthesis system (LI-6400XT; LI-COR, Biosciences, Lincoln, NE, USA). Three plants in each unit were considered for the measurement where data was recorded during the sunny morning hours (8.00–11.30 a.m.). Dark-adapted leaves were deemed for the measurement of CF. The highest efficiency of photosystem 2 (PS-II) was revealed through the ratio of variable fluorescence (Fv) to maximum fluorescence (Fm). The ratio of minimum fluorescence (Fo) to Fm was also calculated using the above-mentioned photosynthetic meter.

The measurement for gas exchange attributed included net photosynthesis and stomatal conductance (gs) were made on a portable photosynthesis system where the following settings were fixed: 30 ± 0.01 °C cuvette temperature, 1.75 cm^−2^ of fixing leaf area, 495 µm of water flow rate and 40% of relative humidity. Likewise, 396 µmol mol^−1^ of the surrounding CO_2_ cons. and 1500 µmol m^−2^ s^−1^ of PAR were also maintained during the measurement. The ratios of photosynthesis to gs and transpiration were considered as intrinsic- (WUEi) and instantaneous-water use efficiency (iWUE), respectively^34,35^.

#### Leaf free proline

Leaf free proline content was determined following standard procedure. Firstly, 0.2 g of fresh leaf samples of both crops were homogenized in 20 mL solution of aqueous sulphosalicylic acid (SA). Next, 4 mL of the filtered homogenized mixture was blended with 4 mL of acid ninhydrin and 4 mL of glacial acetic acid. After incubating the reaction mixture at a high temperature (100 °C) in a water bath, 8 mL of toluene was then added to the vortexing-reacted mixture. Later, proline contents were estimated from the chromophore, according to the above-mentioned protocol.

#### Lipid peroxidation

Thiobarbituric acid-based digestion was made for estimating lipid peroxidation in terms of MDA content, as previously described by Heath and Packer^36^. For that, 2 g of fresh leaf samples of both crops were digested in trichloroacetic acid (10%). Next, the digested sample solution was centrifuged at 15,000×*g* for several minutes at freezing temperature. Later, MDA contents were calculated by considering the supernatant.

#### Antioxidant enzymes

Total soluble proteins in the leaf tissues were estimated by Bradford^37^ method, using fresh leaves samples. The CAT activity was determined following Maehly and Chance^38^. For that, the digestion of fresh leaves samples was made in the H_2_O_2_ and phosphate buffer solutions. Later, the absorbance of the reaction mixture was analyzed at 240 nm wavelength, spectrophotometrically considering the molar extinction coefficient of 36 × 103 mM^−1^ m^−1^. Likewise, the pre-described standard protocol of Giannopolitis and Ries^39^, based on *p*-nitroblue tetrazolium digestion, was followed in order to determine the SOD activity. For that, digestion of fresh leaf samples of both crops was taken place in 10 mL of potassium phosphate buffer solution at chilling temperature. Later, the absorbance was considered at a wavelength of 560 nm at the spectrophotometer, applying the molar extinction coefficient of 4.02 × 103 mol L^−1^ cm^−1^. To determine the POD activity, extraction of fresh leaves sample was made in phosphate buffer solution. Later, the extracted solution was then homogenized in guaiacol. After the addition of H_2_O_2_ solution, POD activity was determined spectrophotometrically, according to the standard etiquette of^40^. Furthermore, APX activity in fresh leaf samples was defined according to Yin et al.^41^, where absorbance was made at 290 nm wavelength.

### Determination of transcript levels of genes

The transcript levels of the genes related to antioxidant activities including glutathione-S-transferase 1 (GST1), glutathione-S-transferase 2 (GST2), glutathione peroxidase 1 (GPX1), phospholipid hydroperoxide glutathione peroxidase 2 (GPX2), glutathione reductase (GR) and glutathione synthetase (GS) were determined according to the protocol of^42^. Firstly, according to the manufacturer’s instructions, total RNA was extracted using the TRIzol reagent. Later, it was treated with RNase-free DNase I (Takara Biotechnology [Dalian] Co., Ltd., Dalian, China) to remove contaminating genomic DNA. About 2 μg of total RNA was used to synthesize the first-strand cDNAs using Super-Script II reverse transcriptase (Invitrogen, Carlsbad, CA, USA). SYBR Premix Ex Taq (Perfect Real Time) kit (Takara Biotechnology [Dalian] Co., Ltd.) on a Light Cycler 480 Real-Time PCR System (Roche Diagnostics Ltd., West Sussex, UK) was used to perform qPCR. The reaction mixture of 20 μL, comprised of 10 μL of SYBR Green Supermix (2×), 1 μL of diluted cDNA, and 0.5 μL of forward and reserve primers, was used. The relative transcript levels were calculated using the 2−ΔΔCt method. The β-actin (GenBank Accession no. AB181991) gene was considered as an internal control. Each data point was expressed as the average ± SD of three independent replicates.

### Statistical analysis

Collected data on physiological and biochemical characteristics were analyzed using Statistix 8.1 software. Two-way ANOVA was applied to evaluate the effects of nitrogen and intercropping treatments. The Tukey HSD test was used to quantify the effects of the treatments i.e., nitrogen and intercropping treatments. Origin-pro software (package 2022) was used to visualize the data graphically. To assess the relationship among tested traits, Pearson correlation analysis was done using the corrplot package.

## Results

### Chlorophyll fluorescence and gas exchange

**Figure 2:**
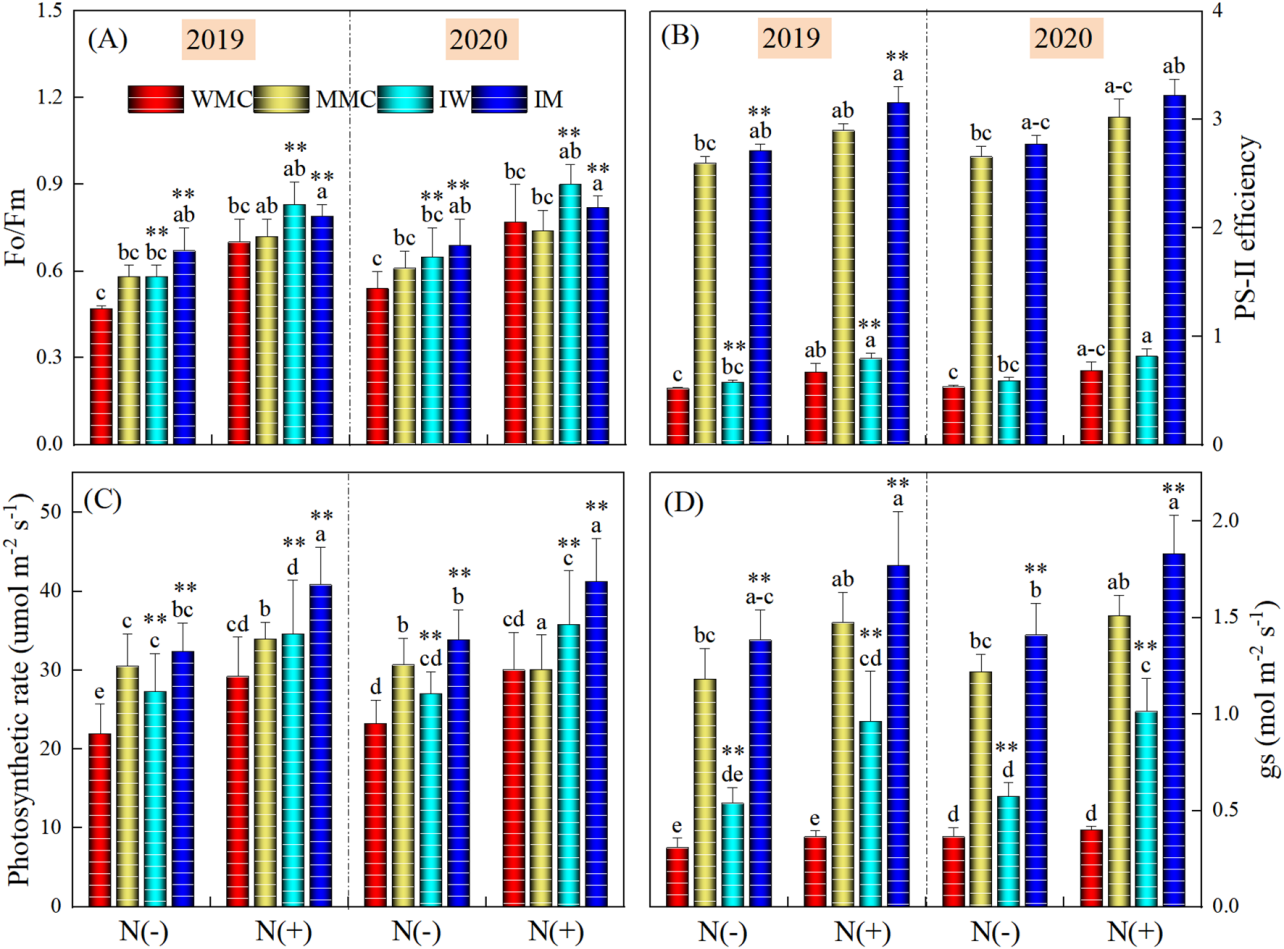
Fo/Fm, PS-II efficiency, photosynthesis, and stomatal conductance (gs) under different intercropping treatments (maize monocrop (MMC), wheat monocrop (WMC), intercropping maize (IM), and wheat (IW) crops) and nitrogen treatments in 2019 and 2020. Different lower-case letters show a significant difference among intercropping treatments under both N rates. “*” above bars indicates a significant difference between the monocropping and intercropping treatments at *P < 0.05; **P < 0.01. *Note*: Fo and Fm indicate the minimum and maximum chlorophyll fluorescence in the dark-adapted state, respectively.

**Figure 3:**
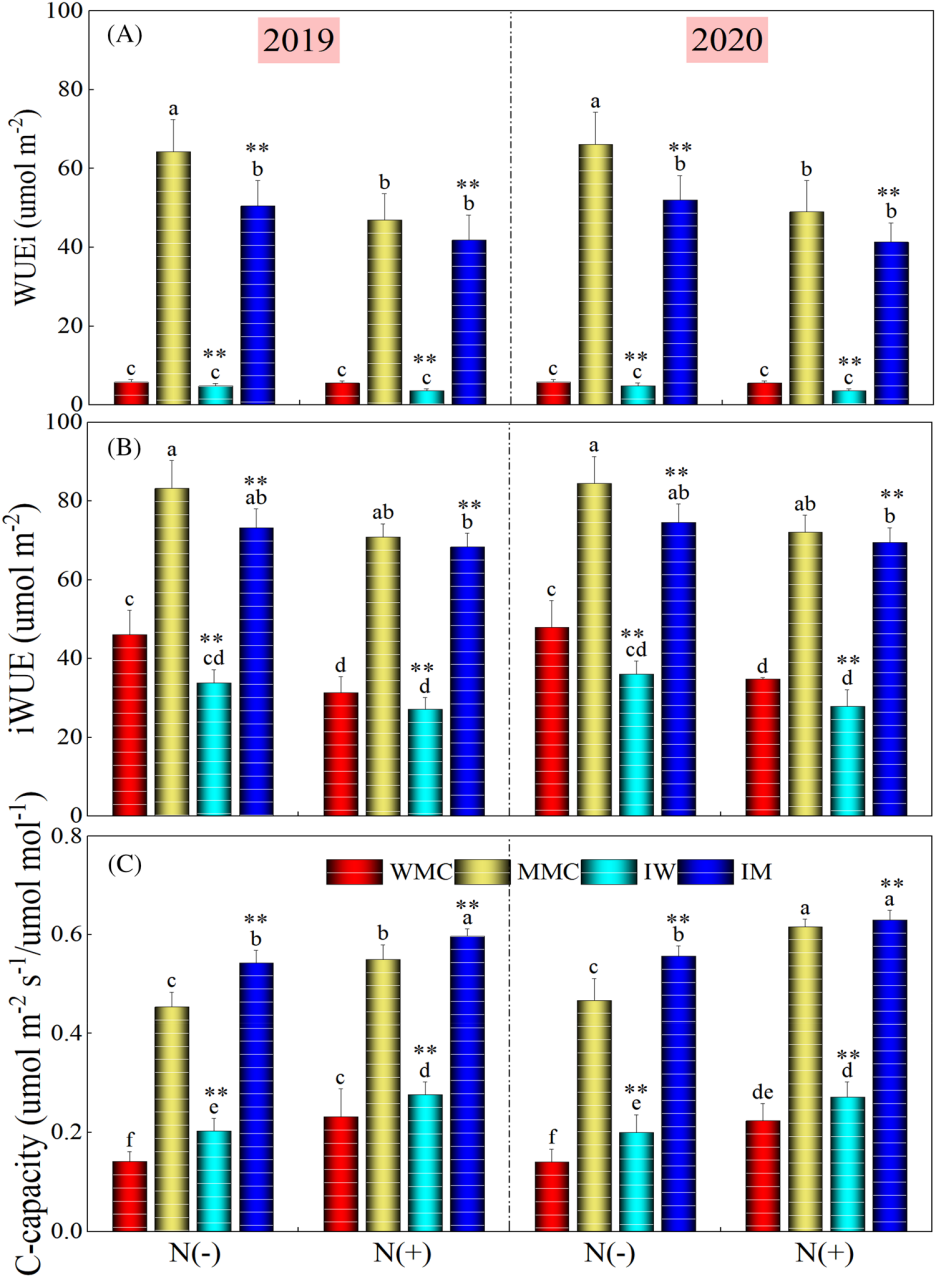
WUEi, iWUE and C-Capacity under different intercropping treatments (maize monocrop (MMC), wheat monocrop (WMC), intercropping maize (IM), and wheat (IW) crops) and nitrogen treatments in 2019 and 2020. Different lower-case letters show a significant difference among intercropping treatments under both N rates. “*” above bars indicates a significant difference between the monocropping and intercropping treatments at *P < 0.05; **P < 0.01. *Note*: WUEi and iWUE indicate the intrinsic and instantaneous water use efficiency, respectively.

Intercropping treatments viz. WMC, MMC, IW, and IM and N application rates significantly affected chlorophyll fluorescence and gas exchange traits (Figs. 2, 3). Compared with wheat (WMC) and maize monocropping (MMC), intercropping-maize (IM) and -wheat rows (IW) recorded significantly (*P* < 0.001) higher values for Fo/Fm, PS-II efficiency, photosynthetic rate, and stomatal conductance under with (Nitrogen+) and with-out N (Nitrogen−) application during both study years. IW and IM increased the Fo/Fm by 22.56 and 14.13% (means of the 2 years), and 18.14 and 10.53% (means of the 2 years) under without- and with-N application, respectively. On average of 2 years, IW and IM increased PS-11 efficiency by 11.20 and 4.57%, and 19.06 and 7.83%, photosynthesis by 20.30 and 8.33%, and 18.84 and 28.75% and stomatal conductance by 66.40 and 16.40%, and 159.24 and 20.66%, compared with WMC and MMC under without- and with-N application, respectively. Comparing two N rates, on average of intercropping treatments, the N addition increased Fo/Fm by 32.05 and 30.15%, PS-II efficiency by 17.34 and 18.08%, photosynthesis by 23.58 and 10.40%, and stomatal conductance by 43.15 and 33.27%, respectively in 2019 and 2020, compared to without N application.

The instantaneous (WUEi) and intrinsic water use efficiency (iWUE), and C-capacity (CC) varied significantly (*P* < 0.001) among intercropping treatments as well as N application rates during both study years. Compared with WMC and MMC, IM and IW depicted a significant (*P* < 0.001) reduction for WUEi and iWUE, and higher values for CC under with and without N application during both study years. On average over 2 years, IW and IM decreased WUEi by 17.13 and 21.33%, 35.45 and 13.38%, iWUE by 25.71 and 11.93%, and 16.81 and 3.61%, compared with WMC and MMC under without- and with-N application, respectively. IW and IM increased CC by 43.53 and 19.57% (means of the 2 years), and 20.53 and 5.32% (means of the 2 years) under without- and with-N application, respectively. Comparing two N rates, on average of intercropping treatments, the N addition decreased WUEi by 21.90 and 22.74%, and iWUE by 16.37 and 15.92%, and increased CC by 23.41 and 27.75%, respectively in 2019 and 2020, compared to without N application.

### Overall water-use efficiency and mean efficiency equivalent ratio

Our results clearly demonstrated that there was a significant difference in water-use efficiency and mean efficiency equivalent ratio among the N treatments, intercropping and experimental years (Table 1). According to our results, there was a significant increase in water-use efficiency and mean efficiency equivalent ratio of intercropping treatments as compared with their respective monocrop treatments. Similarly, N addition (+N) also depicted a significant increase in water-use efficiency and mean efficiency equivalent ratio under all intercropping treatments when compared with control without N application (−N). Moreover, experimental years also affected water-use efficiency and mean efficiency equivalent ratio where there was a significant increase during the second experimental year (Table 1).

**Table 1:** Effect of wheat–maize intercropping system on water use efficiency and mean water use efficiency equivalent ratio during both study years under with- (+ N) and with-out nitrogen (−N) application.

Year	Nitrogen	Intercropping	Water use efficiency (kg ha^−1^ mm^−1^)	Means water use efficiency equivalent ratio
2019	−N	WMC	21.02 d	–
		MMC	23.96 cd	–
		IW	28.45 b	0.99
		IM	29.35 b	1.03
	+ N	WMC	24.56 c	–
		MMC	25.54 c	–
		IW	30.56 ab	0.95
		IM	31.84 a	10.2
2020	−N	WMC	22.03 d	–
		MMC	25.02 cd	–
		IW	29.33 b	0.98
		IM	30.45 b	1.04
	+ N	WMC	25.66 c	–
		MMC	26.74 c	–
		IW	31.76 ab	0.95
		IM	33.04 a	1.05

MMC, Maize monocrop; WMC, wheat monocrop; IM, intercropping maize; IW, intercropped wheat.

### Osmolyte accumulation and MDA contents

**Figure 4:**
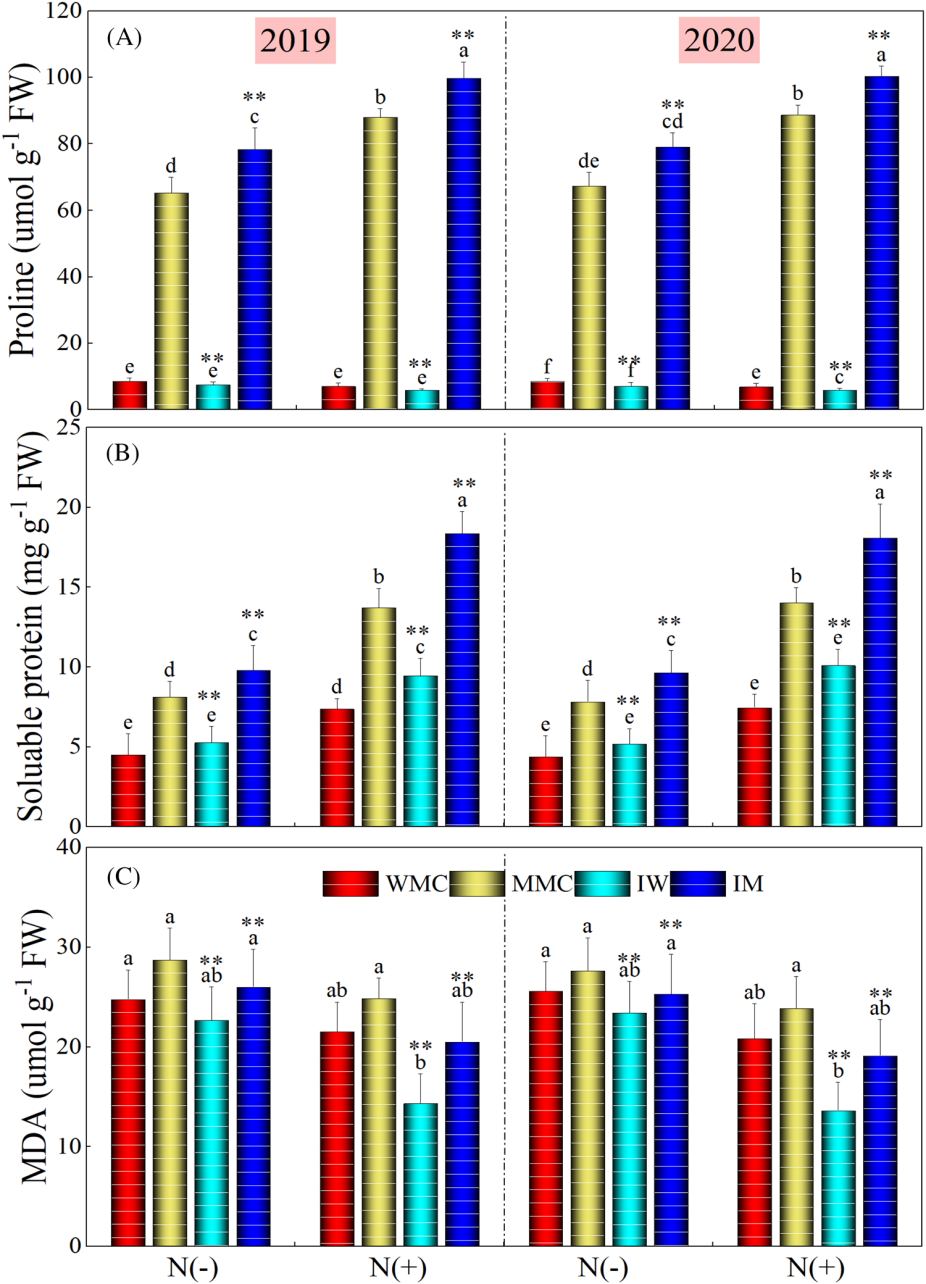
Proline, soluble protein and malondialdehyde (MDA) contents under different intercropping treatments (maize monocrop (MMC), wheat monocrop (WMC), intercropping maize (IM), and wheat (IW) crops) and nitrogen treatments in 2019 and 2020. Different lower-case letters show a significant difference among intercropping treatments under both N rates. “*” above bars indicates a significant difference between the monocropping and intercropping treatments at *P < 0.05; **P < 0.01.

Intercropping and N application rates significantly affected the proline, soluble protein, and MDA contents during both study years. Intercropping treatments increased proline and protein contents under both N rates and experimental years except for IW treatment where a slight decrease was noted for proline content during both study years and for N rates (Fig. 4). IM increased the proline content by 18.75 and 13.21% (means of the 2 years) under without- and with-N applications, respectively, compared with MMC. On average for 2 years, IW and IM increased soluble protein content by 17.53 and 21.73%, and 32.07 and 31.46%, compared with WMC and MMC under without- and with-N application, respectively. There was a significant (P < 0.05) reduction in MDA content for intercropping and N rates during both years. IW and IM decreased MDA content by 8.50 and 8.89% (means of the 2 years), and by 34.01 and 18.60% (means of the 2 years) under without- and with-N application, respectively. Comparing two N rates, on average of intercropping treatments, the N addition increased protein content by 76.43 and 83.72% and decreased MDA content by 20.54 and 24.03%, respectively in 2019 and 2020, compared with the treatment without N application.

### Antioxidant enzymes

**Figure 5:**
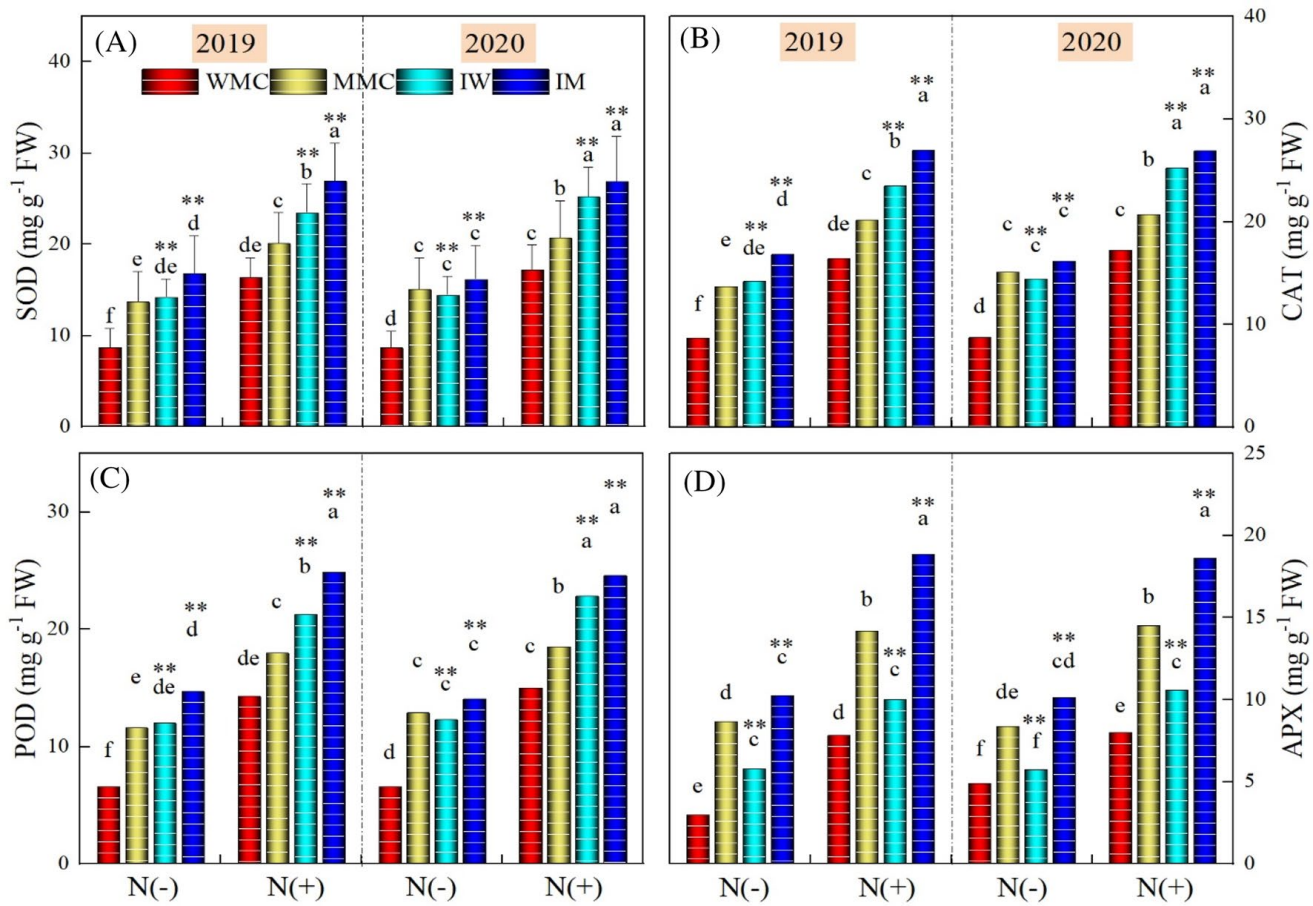
SOD, CAT, POD and APX activity under different intercropping treatments (maize monocrop (MMC), wheat monocrop (WMC), intercropping maize (IM), and wheat (IW) crops) and nitrogen treatments in 2019 and 2020. Different lower-case letters show a significant difference among intercropping treatments under both N rates. “*” above bars indicates a significant difference between the monocropping and intercropping treatments at *P < 0.05; **P < 0.01. *Note*: CAT, SOD, POD and APX indicate catalase, superoxide dismutase, peroxidase and ascorbate peroxidase activity, respectively.

The activities of all studied antioxidant enzymes varied significantly among the intercropping treatments and N rates. Intercropping and N application significantly (*P* < 0.001) improved the activities of SOD, CAT, POD, and APX enzymes during both study years (Fig. 5). On average over 2 years, IW and IM increased SOD activity by 64.90 and 14.88%, 45.15 and 32.01%, CAT activity by 64.56 and 14.89%, and 44.89 and 31.87%, POD activity by 84.89 and 17.90%, and 50.48 and 35.54%, and APX activity by 15.72 and 20.16%, and 30.16 and 30.54%, compared with WMC and MMC under without- and with-N application, respectively. When comparing two N rates, on average of intercropping treatments, the N addition increased the SOD activity by 62.60 and 65.64%, CAT activity by 62.68 and 65.39%, POD activity by 74.90 and 76.52%, and APX activity by 70.96 and 77.58%, respectively in 2019 and 2020, compared with the treatment without N application (Fig. 5).

### Person correlation

**Figure 6:**
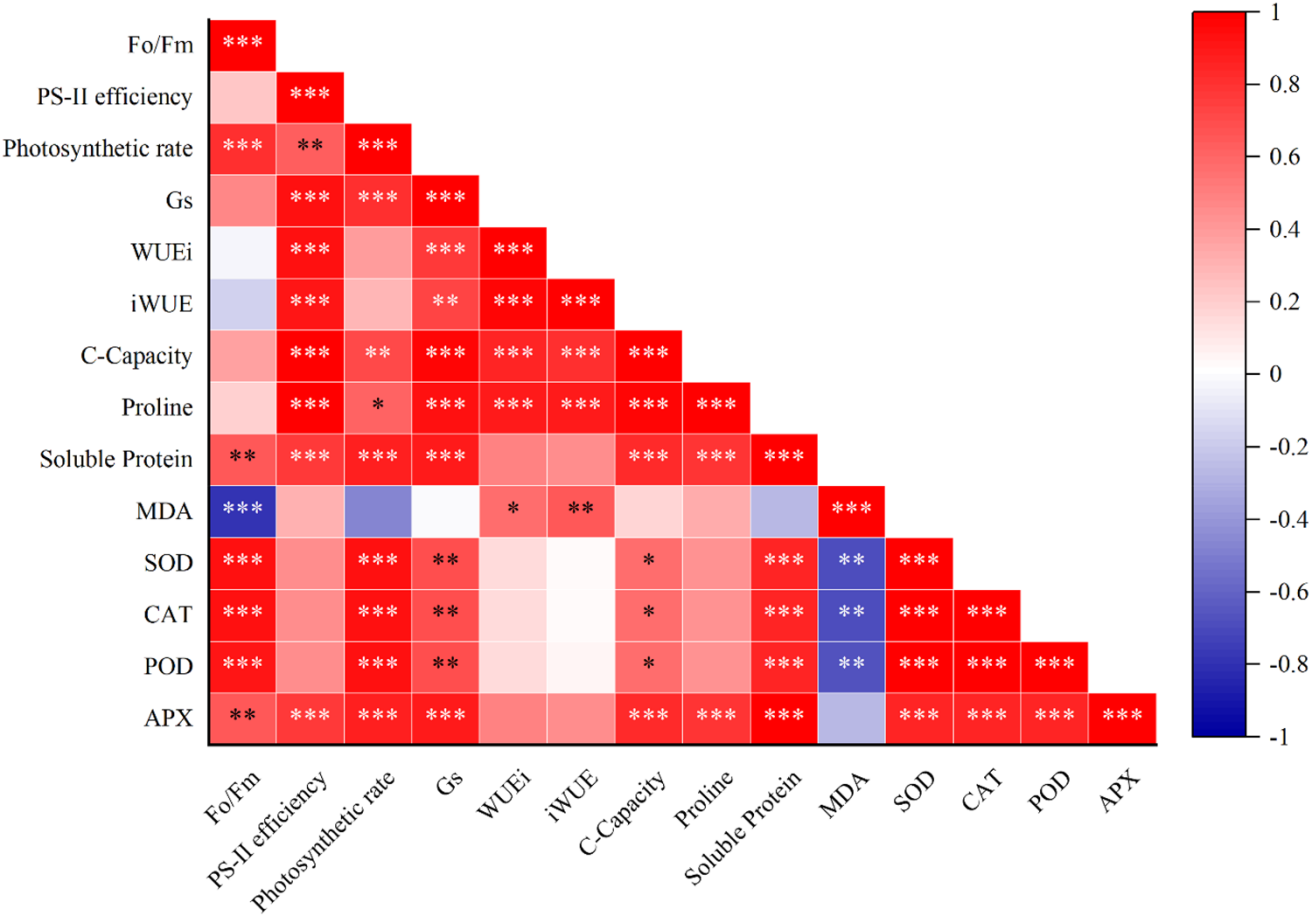
Pearson correlation coefficient of chlorophyll fluorescence, gas exchange attributes and antioxidants under different intercropping treatments and N application rates (n = 3). Fo and Fm indicate the minimum and maximum chlorophyll fluorescence in the dark-adapted state, respectively; gs donates stomatal conductance; WUEi and iWUE indicate the intrinsic and instantaneous water use efficiency, respectively; MDA shows malondialdehyde; CAT, SOD, POD and APX indicate catalase, superoxide dismutase, peroxidase, and ascorbate peroxidase activity, respectively.

The Fo/Fm had a strong positive correlation with photosynthesis, SOD, CAT, and POD, and had a strong negative correlation with MDA. Likewise, PS-II had a strong positive correlation with Gs, WUEi, iWUE, C-Capacity, proline, protein, and APX. Photosynthesis had a strong positive correlation with Gs, protein, SOD, POD, CAT, and APX. GS had a strong positive correlation with WUEi, C-Capacity, proline, protein, and APX, and had a positive correlation with iWUE, SOD, CAT, and POD. WUEi and iWUE had a strong positive correlation with C-Capacity, proline, and with each other, and had a positive correlation with MDA. C-Capacity and proline had a strong positive correlation with protein, APX, and each other. The soluble protein content had a strong positive correlation with antioxidant enzymes and a negative correlation with MDA. Likewise, MDA had a negative correlation with SOD, POD, and CAT. While, SOD, CAT, POD, and APX had a strong positive correlation with each other (Fig. 6).

### Transcript level

**Figure 7:**
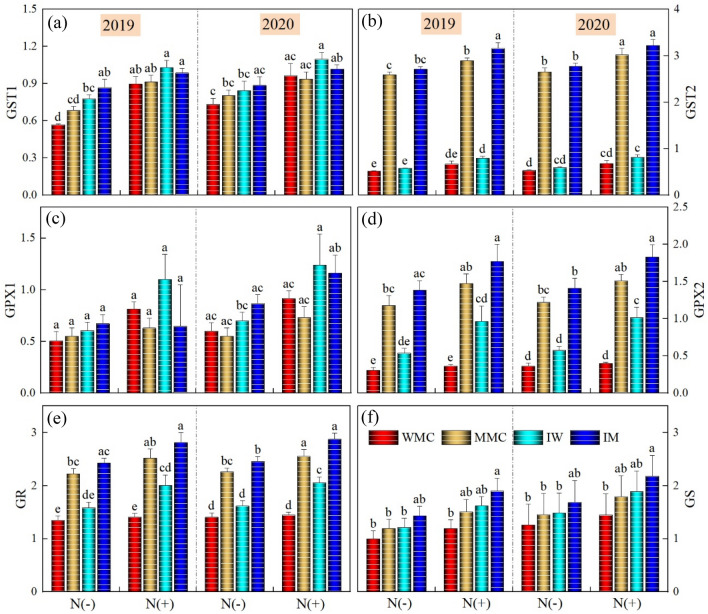
Effects of intercropping treatments and N rates on transcript levels of the six genes encoding ASA-GSH cycle enzymes in root of both wheat and maize crops during both years. Transcripts were analyzed by qPCR using Actin gene as internal control. The three seedlings were collected in one replication and three independent biological replications were performed. Each value is the mean ± standard error of three independent measurements. Intercropping treatments were: (maize monocrop (MM), wheat monocrop (WM), intercropping maize (IM), and wheat (IW) crops). Different lower-case letters show a significant difference among intercropping treatments under both N rates.

**Figure 8:**
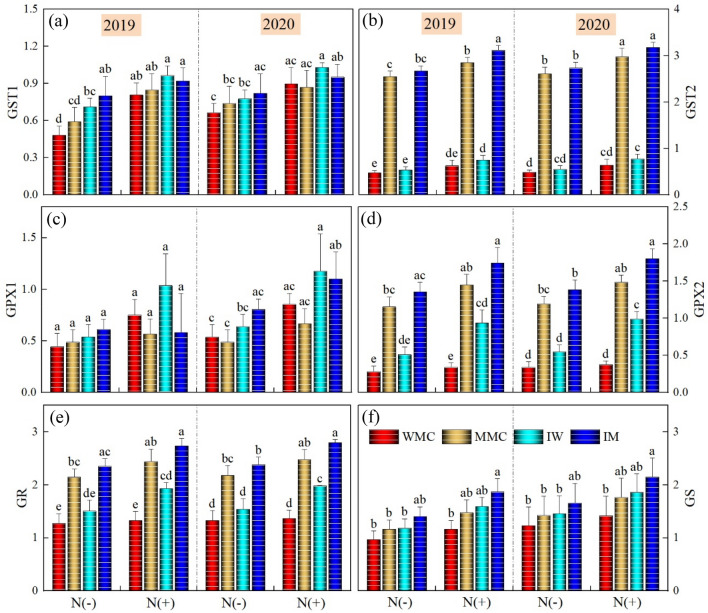
Effects of intercropping treatments and N rates on transcript levels of the six genes encoding ASA-GSH cycle enzymes in leaves of both wheat and maize crops during both years. Transcripts were analyzed by qPCR using Actin gene as internal control. The three seedlings were collected in one replication and three independent biological replications were performed. Each value is the mean ± standard error of three independent measurements. Intercropping treatments were: (maize monocrop (MM), wheat monocrop (WM), intercropping maize (IM), and wheat (IW) crops). Different lower-case letters show a significant difference among intercropping treatments under both N rates.

In this work, GST1, GST2, GPX1, GPX2, GR, and GS transcript levels were measured using qPCR. The Actin gene was kept as the internal control in both leaf and root tissues of wheat and maize seedlings under different intercropping and N treatments (Fig. 7). The effect of years was nonsignificant for all genes. Intercropping and N treatments significantly affected the relative expression of GST1, GST2, GPX2, and GR whereas there was a non-significant influence on GPX1 and GS both in root and leaves. Among intercropping treatments, intercrops depicted somehow higher expression levels in both root and leaves as compared to monocrops where maize intercropping depicted higher values than the respective monocrop. Among N treatments, the N addition recorded a significantly higher expression level than the control treatment without N addition (Fig. 8).

## Discussion

The results supported the hypothesis that intercropping practice is highly effective in improving resource use efficiency and overall crop performance, particularly in rainfed areas. Intercropping treatments significantly improved the chlorophyll fluorescence, and gas exchange traits such as Fo/Fm, PS-II, photosynthesis, stomatal conductance, and C-Capacity when compared with monocropping treatments (Figs. 2, 3). A significant decrease in water use efficiencies (WUEi and iWUE) and MDA contents were noted for intercropping treatments. However, intercropping treatments i.e., IW and IM significantly improved the antioxidant enzyme activities including CAT, SOD, POD, and APX (Fig. 5). Under field conditions, mostly in arid areas, plants endure moisture stress when the required water levels are unobtainable in the rhizosphere, particularly under high evapotranspiration conditions^43,44^. According to previous studies, osmotic stress under moisture stress conditions affected the plant’s physiological and biochemical traits^45,46^ as has been depicted in this work (Figs. 2, 3, 4, 5, 6). Under extreme dryness, osmotic stress causes stomatal closure, impairs mitosis, and losses of turgor which results in a significant reduction in physiological events including stomatal conductance and photosynthesis^47^ as recorded in this study (Fig. 2). Osmotic conditions also disrupt stomatal conductance owing to agitated plant-water relations and reduced activities of photosynthetic pigments and synthesis of phytohormones including ABA that triggers stomatal closure and reduces intercellular CO_2_ levels^48^. Nonetheless, intercropping treatments significantly improved the physiological traits of both crops when compared with their respective monocropping treatments under rainfed conditions (Figs. 2, 3). Simultaneously cultivation of C_3_ and C_4_ crops in the same field alters the ventilation and provides better conditions for light interception^49^. As reported previously, compared with C_3_ crops, C_4_ had more height and showed higher light saturation levels for better photosynthesis^50,51^. Moreover, previous studies have demonstrated that the combination of C_3_ and C_4_, as high-position crops, provided compensation while capturing the sunlight for better photosynthesis^52,53^. It is generally claimed that chlorophyll fluorescence is an important indicator to draw the relationships between the photosynthetic levels and the surrounding environments; this phenomenon is critical in estimating the efficacy of intercepted light, and the absorption and distribution of captured light during photosynthesis^54^. Estimating the efficacy of PSII and Fo/Fm is critical to determine the plants’ photosynthetic efficiency^55^. In this work, our results demonstrated that intercropping treatments significantly improved the efficacy of PSII, Fo/Fm, and overall photosynthesis when compared with respective monocropping treatments. Higher photosynthesis under intercropping might be associated with better activities of photosynthesis enzymes, as previously reported by^51^. In C_4_ plants, PEPC and RuBisCO enzymes play essential roles during the process of carbon assimilation, in this way can determine the efficacy of leaf photosynthesis^51^. According to previous reports, RuBisCO plays critical roles in carboxylation and oxygenation during photosynthesis; this enzyme is a key driver of photorespiration^56,57^. Furthermore, it is also well demonstrated that the PEPC enzyme is involved in the fixation of primary carbon dioxide in C_4_ plants^58^.

Under moisture stress, plants also experienced numerous changes at biochemical levels including the excessive production of ROS that results in lipid peroxidation and membrane damage^59^. Malondialdehyde, a product of lipid peroxidation, is demonstrated responsiveness to oxidative stress^60^. Consequently, malondialdehyde contents are used as an indicator of plant tolerance to abiotic stresses^61^. In this work, intercropping treatments depicted significantly lower values of MDA content showing higher tolerance to water deficit stress. Furthermore, as an adaptive response, numerous antioxidative enzymes such as SOD, POD, CAT, and APX are also produced in plants to detoxify the ROS-induced effects^62,63^. In this work, our results demonstrated that intercropping treatments significantly increased the activities of antioxidant enzymes as compared with their respective monocropping treatments (Fig. 4). Similar to our results, Zheng et al.^51^ reported that intercropping treatments substantially increased the activities of SOD, POD, CAT, and APX enzymes and decreased lipid peroxidation by decreasing the MDA activities. Recently, Cui et al.^64^ studied the influence of intercropping treatments on antioxidant enzyme activities and reported that these treatments decreased the toxic effects of oxidative stress by increasing the activities of antioxidant enzymes. Antioxidative enzymes such as CAT, SOD, APX, and POD, are proficient to sustain ROS levels and counteract the plants from lipid peroxidation (membrane damage). Indeed, under high ROS conditions, plants can protect cells from oxidative stress by inducing a strong antioxidative defense system^65,66^. Previously, Cho and Seo^67^ proposed that antioxidant enzymes sustain and modulate the H_2_O_2_ levels for signaling during metabolic alterations under stressful conditions. In this work, IM and IW treatments significantly improved the activities of antioxidants in both maize and wheat crops, compared with monocropping treatments. Moreover, antioxidant enzymes had a negative correlation with the MDA content, showing that IM and IW treatments can improve crop performances and alleviate oxidative damage by improving the activities of antioxidant enzymes under water stress.

GSH and ASA are major non-enzymatic antioxidants that play significant roles in the scavenging of ROS^68^. Overexpression of the genes related to non-enzymatic antioxidants confers enhanced tolerance to abiotic stresses in crop plants^69^. Transcriptional analysis helps to quantify the changes in transcript levels of genes that are involved in the regulation of metabolism. In this work, the expression levels of six genes encoding ASA-GSH cycle enzymes were determined in wheat–maize seedlings exposed to various intercropping treatments and N application. According to our results, in root and leaf tissues of both wheat and maize seedlings under intercropping treatments and N rates, the transcript profiles of ASA-GSH synthesis-related genes varied and intercropping treatment markedly enhanced the expression of these genes. In line with these results, Li et al.^70^ also reported enhanced expression levels of various genes involved in regulating the activities of enzymatic and non-enzymatic antioxidants. Similar was also reported by Wei et al.^71^ who identified various genes regulating the activities of non-enzymatic antioxidants.

Under water stress, to maintain cellular hydration, through osmotic adjustment, crop plants also accumulate solutes that work as osmolytes and play critical roles in the protection of cellular structure^72,73^. It has been reported that intercropped crops can accumulate more solutes for their survival under moisture stress than monocrops^74^. Similar was found in this work in which intercropping treatments significantly improved proline and protein contents in both crops (Fig. 5). Furthermore, a strong positive correlation among proline, protein, and antioxidant enzymes also showed that these enzymes and solutes accumulation help to sustain intercropping crops under moisture stress conditions (Fig. 6). The synthesis and accumulation of proline also take place in plants to induce tolerance to water stress^75^. It has been well established that proline also owns antioxidative belongings and protects the plant cells from dehydration when acting as chaperones to shield the macromolecule assembling^76^. The radical scavenger properties of proline are also well demonstrated in previous studies^77,78^.

## Conclusion

Our results indicated that both maize and wheat crops when grown under intercropping system performed better under rainfed conditions than their respective monocrops. Intercropping treatments with significantly higher proline and protein contents, better chlorophyll fluorescence, the activities of antioxidative enzymes such as SOD, POD, CAT, and SOD, and lower MDA levels were better able to endure their growth and development under moisture deficit conditions. Use of these traits i.e., chlorophyll fluorescence, antioxidative enzyme activities, and osmolytes accumulation will be of interest in future breeding programs to produce drought-tolerant genotypes, particularly for rainfed conditions.

## Data Availability

All obtained data is enclosed with this manuscript.
